# Supplementary material for: EndoGeneAnalyzer: A tool for selection and validation of reference genes
Source: PLoS One. 2024 Apr 3;19(4):e0299993. doi: 10.1371/journal.pone.0299993 (PMC10990236; doi:10.1371/journal.pone.0299993)
Supplement: Attachment — Submitted filename: Review of EndoGeneAnalyzer R1.docx [file pone.0299993.s002.docx]

Well done to the authors for addressing my comments, I think the improvements to the app will make it much easier to use. My remaining comments are largely minor and can be easily addressed.

**Comments** (all line numbers relate to the tracked changes version of the MS)

Line 31 – change to ‘tool to assist’

Line 32 – lower case Q

Line 46 – remove The

Line 63 – only as it pertains to gene expression studies. RT-qPCR is used for many other purposes as well.

Lines 65-67 – This sentence should not be a paragraph, instead, make it the first sentence of the previous paragraph.

Lines 72-74 – cite Chapman and Waldenström for this point (stability of ref genes within study context)

Line 206 – remove second ‘the’ on this line

Some of the wording in the tutorial section of the app needs proof-reading (perhaps by a native English speaker) for clarity.

Have you considered having an option for Bonferroni (or other method) correction for multiple comparisons in the Differential Expression Analysis?
