# Supplementary material for: EndoGeneAnalyzer: A tool for selection and validation of reference genes
Source: PLoS One. 2024 Apr 3;19(4):e0299993. doi: 10.1371/journal.pone.0299993 (PMC10990236; doi:10.1371/journal.pone.0299993)
Supplement: Attachment — Submitted filename: Response to Reviewers.docx [file pone.0299993.s003.docx]

**RESPONSE LETTER TO THE REVIEWERS**

Dear reviewer, my co-authors and I would like to thank you for the suggestions made during this high-quality review and then we present the answer to the questions.

We inform that with the reviews and suggestions, we were able to improve the idea presented by our work and we appreciate the opportunity. We hope this review has left the article suitable for publication in this high-impact and prestigious journal.

Kind Regards.

Reviewer #1: Well done to the authors for addressing my comments, I think the improvements to the app will make it much easier to use. My remaining comments are largely minor and can be easily addressed.

Comments (all line numbers relate to the tracked changes version of the MS)

Line 31 – change to ‘tool to assist’

R=Thank you for your suggestion! The change to 'tool to assist' has been implemented.

Line 32 – lower case Q

R=Thank you for your suggestion! The change to lower case 'Q' on Line 32 has been implemented

Line 46 – remove The

R= Thank you for your suggestion! We remove ‘The’ on Line 46

Line 63 – only as it pertains to gene expression studies. RT-qPCR is used for many other purposes as well.

R= Thanks for your suggestion! We have clarified the sentence in Line 63 to explicitly state that in the study RT-qPCR refers only to gene expression studies.

Lines 65-67 – This sentence should not be a paragraph, instead, make it the first sentence of the previous paragraph.

R=Thank you for your input! We have reorganized the text as per your suggestion. The content from Lines 65-67 is now the first sentence of the preceding paragraph.

Lines 72-74 – cite Chapman and Waldenström for this point (stability of ref genes within study context)

R=Thank you for your suggestion! We cite Chapman and Waldenström on lines 72-74

Line 206 – remove second ‘the’ on this line

R= Thank you for your suggestion! We remove second ‘the’ on Line 206

Some of the wording in the tutorial section of the app needs proof-reading (perhaps by a native English speaker) for clarity.

R= Thank you for pointing it out, we reviewed the tutorial section wording.

Have you considered having an option for Bonferroni (or other method) correction for multiple comparisons in the Differential Expression Analysis?

R=After ANOVA, Tukey's post hoc test is used for multiple comparisons and already provides the adjusted p-value. Similarly, following Kruskal-Wallis, the Dunn post hoc test is applied for multiple comparisons, and the p-values are adjusted using the Benjamini-Hochberg method. We added this information at the tutorial section.

Reviewer #2: The revised manuscript and corresponding application have undergone significant improvements; however, some of my previous concerns were not fully addressed.

Comments on the manuscript:

I appreciate the creation of a comprehensive tutorial page for your tool. Nevertheless, some information is missing in the manuscript. The method for outliers removal, a previous concern of mine, is now clearly described in the tutorial page but not adequately addressed in the manuscript (lines 143-144). Additionally, the new functionality of restoring outliers is not mentioned in the manuscript.

R= Thank you for your feedback. We appreciate your acknowledgment of the comprehensive tutorial and note your concerns. We will enhance the manuscript by providing a more detailed explanation of the outliers removal method (lines 143-144) and include information about the new functionality for restoring outliers.

While the authors provided a conclusion for their manuscript, the sentences are overly complicated, hindering understanding, and contain grammar mistakes. The conclusion should also mention the differential expression analysis as one of the additional features of this tool. Please delete the last sentence.

Regarding the grammar and flow of the text, I recommend having a native speaker review and edit the whole manuscript.

R= We've made the changes as requested, and the manuscript has been sent for translation by native speakers. Regarding the conclusion, the sentences were overly complex, making it difficult to understand, and there were grammar mistakes. We've also added a mention of the differential expression analysis as one of the additional features of the tool. However, we've removed the last sentence.

Comments on the figures:

Fig 1: Correct the name of third step from: "Gene Reference Sample" to "Gene Reference Samples".

R= Thank you for your feedback. We have made the correction in Fig 1 by updating the name of the third step from 'Gene Reference Sample' to 'Gene Reference Samples.

Fig 2: Check the description of this figure in the manuscript. Change "Ct" to "Cq".

R= Thank you for your observation. We have revised the description of Fig 2 in the manuscript, changing 'Ct' to 'Cq' as per your suggestion."

Fig 4: The values in the figure are currently presented only in black and white, despite the description referencing values highlighted in red. To ensure accuracy, update the figure to include the specified coloration. Additionally, align the numbering format consistently between the figure and its description (e.g., use 1, 2, 3 in both). Furthermore, incorporate the respective p-values associated with the color change into the figure description. This ensures that the significance of the color variation is appropriately communicated in the context of the data presented.

R= Thank you for your observation. For Figure 4, we've updated the coloration to include the specified red highlighting to match the description accurately.

Other minor comments:

Lines 32 + 41: According to the MIQE guidelines (Nomenclature 1.1) RT-qPCR stands for "reverse transcription quantitative real-time polymerase chain reaction".

R= Thank you for your suggestion. We have implemented the change, and the text now correctly states: 'RT-qPCR stands for reverse transcription quantitative real-time polymerase chain reaction.

Line 55: Explain the meaning of Cq here.

R=Thank you for your feedback. We have addressed the request on Line 55 and provided an explanation for the meaning of Cq.

Lines 58 – 61: Check the grammar and clarity of this sentence. Firstly, correct "data normalization process to expression comparison" to "data normalization process for expression comparison". Secondly, consider making the last addition a standalone sentence: "The normalization of data using reference genes involves correcting errors that arise from the initial concentration of RNA/cDNA.

R= Thank you for your suggestions. We have addressed the concerns in Lines 58-61. Firstly, we corrected "data normalization process to expression comparison" to "data normalization process for expression comparison." Additionally, we separated the last addition into a standalone sentence, which now reads: 'The normalization of data using reference genes involves correcting errors that arise from the initial concentration of RNA/cDNA.'

Line 67: Check references on this line. Either put respective reference after mentioned gene or put all references at the end of the sentence into single brackets.

R= Thank you for your guidance. We have revised Line 67 by placing all references at the end of the sentence within single brackets.

Line 128: Delete comma in this sentence.

R= Thank you for your suggestion. The comma has been deleted in this sentence.

Line 150: Correct "Reference of genes by group" to "Gene Reference by group" to be consistent with the tool.

R=Thank you for your feedback. The change "Reference of genes by group" has been corrected to "Gene Reference by group" for consistency with the tool.

Lines 163 + 165: Check equations, especially the summation symbols and remove word "group" from the summation symbol, it is redundant.

R=Thank you for your feedback. Equations have been checked, and the word "group" has been removed from the summation symbol as it was redundant.

Line 164: Remove this line.

R=Thank you for your suggestion. This line has been removed.

Line 200: Change "tutorial" to "Tutorial".

R=Thank you for your suggestion. We change "tutorial" to "Tutorial."

Line 226: Change "meanRef" to "MeanRef".

R=Thank you for your suggestion. We change "meanRef" to "MeanRef."

Line 269: Check references on this line. Put all references at the end of the sentence into single brackets.

R= Thank you for your suggestion. References on this line have been checked, and all references at the end of the sentence are now in single brackets.

Comments on the App:

Regarding the "Update Target Gene": I was testing your tool and I have selected several genes and pressed the "Update Target Gene" button. After that I tried to unselect any targeted genes and press this button again, but nothing has changed and targets stayed the same. I do not think this a desirable feature.

R= Thank you for your careful review. We hadn't noticed this misbehavior. The issue has been fixed.

Regarding this concern "Clarify the purpose and effects of the "Select one or more Genes" option in the tool.": Although the tutorial and the tool provide an explanation now, I would recommend to change the title "Select one or more Genes" to "Selected Reference Genes" to be more intuitive.

R= Suggestion accepted. Thank you.

On the "Gene Reference Analysis" tab change all occurrences of "Normfinder" to "NormFinder".

R= Suggestion accepted. Thank you.

On the "Tutorial" tab:

Point 1: Change "meanCT" to "mean Cq".

R= Thank you for your careful review.Suggestion accepted.

Point 2,3 and 5: Corresponding figures have on y-axis "meanCT", change it to "meanCq".

R= Thank you for your careful review.Suggestion accepted.

Point 5.2: Here, you mention "2^-ΔCq" and in the manuscript "2-ΔΔCq" formula.

R= The term 2^-ΔCq is employed for estimating expression levels, while 2^-ΔΔCq is utilized for estimating fold-change. In this section, where we elucidate the estimation of expression levels, the formula was applied within the appropriate context.

Please correct the Tutorial page for consistency and check grammar.

R=Thank you for your careful evaluation. Suggestion accepted. The tool page and manuscript have been revised.

Finally, let me make two more suggestions for the tool.

Regarding the "Gene Reference Samples" tab: I would recommend to add a short description to outlier removal methods, so user can read the description here and decide which method to use. I really like the added description for the "Gene Reference Analysis" tab, which made the analysis easier. I believe that this tab should be treated in the same way and this addition would make it more user-friendly.

R= Thank you for your careful review.Suggestion accepted.

Create a new tab in the tool called "About". There, provide your contact information for the users as you have also added a warning to the upload page saying "An error has occurred. Check your logs or contact the app author for clarification." but contact information is missing. I would also recommend to provide a link to GitHub page with your code and keep your GitHub page with codes updated. Users can leave feedback there, if they run into some problems, or if they request a new feature to be added.

R=We have implemented the "About" tab as recommended, incorporating a concise tool description, contact and support details, GitHub link, information about participants and funding partners. Additionally, upon acceptance, we will include the publication link.

In summary, commendable progress has been made in refining the manuscript and its corresponding application; however, there are still areas that require attention. Focusing on specific concerns, particularly in enhancing clarity, consistency, and functionality, will undoubtedly contribute to the continuous improvement and effectiveness of the tool. The commitment to elevate the overall quality and user experience of both the manuscript and the application is evident, and I look forward to seeing the implementation of these valuable improvements for the benefit of users and the advancement of your tool within the scientific community.
